# Rationally evolving tRNA^Pyl^ for efficient incorporation of noncanonical amino acids

**DOI:** 10.1093/nar/gkv800

**Published:** 2015-08-06

**Authors:** Chenguang Fan, Hai Xiong, Noah M. Reynolds, Dieter Söll

**Affiliations:** 1 Department of Molecular Biophysics and Biochemistry, Yale University, New Haven, CT 06520-8144, USA; 2 Department of Chemistry, Yale University, New Haven, CT 06520-8144, USA

## Abstract

Genetic encoding of noncanonical amino acids (ncAAs) into proteins is a powerful approach to study protein functions. Pyrrolysyl-tRNA synthetase (PylRS), a polyspecific aminoacyl-tRNA synthetase in wide use, has facilitated incorporation of a large number of different ncAAs into proteins to date. To make this process more efficient, we rationally evolved tRNA^Pyl^ to create tRNA^Pyl-opt^ with six nucleotide changes. This improved tRNA was tested as substrate for wild-type PylRS as well as three characterized PylRS variants (N^ϵ^-acetyllysyl-tRNA synthetase [AcKRS], 3-iodo-phenylalanyl-tRNA synthetase [IFRS], a broad specific PylRS variant [PylRS-AA]) to incorporate ncAAs at UAG codons in super-folder green fluorescence protein (sfGFP). tRNA^Pyl-opt^ facilitated a 5-fold increase in AcK incorporation into two positions of sfGFP simultaneously. In addition, AcK incorporation into two target proteins (*Escherichia coli* malate dehydrogenase and human histone H3) caused homogenous acetylation at multiple lysine residues in high yield. Using tRNA^Pyl-opt^ with PylRS and various PylRS variants facilitated efficient incorporation of six other ncAAs into sfGFP. Kinetic analyses revealed that the mutations in tRNA^Pyl-opt^ had no significant effect on the catalytic efficiency and substrate binding of PylRS enzymes. Thus tRNA^Pyl-opt^ should be an excellent replacement of wild-type tRNA^Pyl^ for future ncAA incorporation by PylRS enzymes.

## INTRODUCTION

In the past few years, more than 100 noncanonical amino acids (ncAAs) have been incorporated into proteins in both prokaryotic and eukaryotic organisms. Genetic code expansion is one of the most powerful approaches (1–4). It uses an orthogonal pair of aminoacyl-tRNA synthetase (aaRS) and tRNA to direct the incorporation of a ncAA with a novel functional group in response to a specific codon at the precisely controlled position in the protein of interest. By this approach, scientists have added new chemistries into different organisms: (i) photo-crosslinkers for mapping weak, transient or pH sensitive protein interactions; (ii) post-translational modifications such as acetylation and phosphorylation for regulating biological processes or identifying modifying enzymes; (iii) photo-caged UAAs for controlling the signaling and channeling by light; (iv) biophysical probes and labels for providing exquisite time-resolved insights into how proteins respond to stimuli (5–7). Combined with other techniques such as imaging, single-molecule and mass spectrometry (MS), genetic code expansion provides a powerful platform for solving biological problems.

As the key components of the genetic code expansion strategy, various orthogonal aaRS•tRNA pairs were developed. Among them, the most frequently used ones were an evolved orthogonal pair based on the *Methanocaldococcus jannaschii* tyrosyl-tRNA synthetase and its cognate tRNA (8), as well as the natural orthogonal pair of Pyrrolysyl-tRNA synthetase (PylRS) and its cognate tRNA, tRNA^Pyl^ from *Methanosarcinaceae* species (9). The PylRS•tRNA^Pyl^ pair exhibits several unique features. Firstly, it is orthogonal across different organisms including *E. coli*, yeast, mammalian cells and animals (10–13). Secondly, it does not use any one of the 20 canonical amino acids, and thus there is no need to eliminate its natural substrate specificity before evolving for a new ncAA. Moreover, besides a broad substrate tolerance with Pyl analogs (14), the evolved PylRS variants also showed a high flexibility with Lys, Phe, Tyr analogs and even α-hydroxy acids (9). Last but not least, PylRS and its variants have low selectivity toward the tRNA anticodon (15). It has been used for incorporating various ncAAs toward stop codons, quadruple codons and certain sense codons (16–19).

Currently, one challenge for genetic code expansion is the relatively poor catalytic efficiency of evolved orthogonal tRNA synthetases, usually with a reduction in aminoacylation efficiency (*k*_cat_/*K*_M_) by a factor of ∼1000 (2). For example, PylRS variants have been selected for incorporating AcK into proteins to facilitate studies of protein acetylation, an important post-translational modification across different organism (11,20,21). Previous studies showed that the catalytic efficiency of AcKRS was >6000 times less than that of wild-type PylRS (22), and further optimization of these AcKRS•tRNA^Pyl^ pairs is highly desirable, especially for incorporating AcK into proteins at multiple positions. Here, we rationally evolved tRNA^Pyl^ for improved AcK incorporation into proteins in *E. coli*. Different from previous studies which randomized residues in the anticodon stem of tRNA^Pyl^ for quadruplet codons suppression (17,18), we focused on the residues in the acceptor and T stems which were expected to interact with elongation factor-Tu (EF-Tu) (23–26) because tRNA^Pyl^ comes from archaea, possibly with a non-optimal binding to *E. coli* EF-Tu. We demonstrated that the AcKRS•tRNA^Pyl-opt^ pair increased multiple AcK incorporation into three different proteins. For broader applications, tRNA^Pyl-opt^ also improved the incorporation of six other ncAAs by wild-type PylRS and two different PylRS variants. Kinetic analyses and northern blotting indicated tRNA^Pyl-opt^ will be an excellent replacement of wild-type tRNA^Pyl^ in any future incorporation strategy.

## MATERIALS AND METHODS

### General molecular biology and plasmid constructions

Oligonucleotide synthesis, DNA sequencing and LC-MS/MS were performed by the Keck Foundation Biotechnology Resource Laboratory at Yale University. The amino acids in this study were purchased from Sigma-Aldrich or ChemImpex. *E. coli* TOP10 cells (Life Technologies) were used for general cloning and screening experiments. Plasmids: The genes of tRNA synthetases and tRNA^Pyl^ were cloned by PCR from laboratory inventory and inserted into the *pTech* plasmid. The tRNA synthetase genes were placed under the constitutive *lpp* promoter, while the tRNA gene was placed under the constitutive *proK* promoter. The gene of codon-optimized super-folder green fluorescent protein (sfGFP) with C-terminal His_6_-tag was cloned into the *pBAD* plasmid, and placed under the control of the inducible arabinose promoter. All the cloning experiments were performed by using the Gibson Assembly kit (New England Biolabs). The mutations of tRNA and stop codons in protein genes were made by the QuikChange II mutagenesis kit (Agilent Life Sciences). The SDS-PAGE gel is 4–20% gradient gel purchased from Bio-Rad.

### sfGFP readthrough assay

*E. coli* TOP10 cells were used to express sfGFP and its variants. The strains harboring sfGFP reporters as well as tRNA synthetases and tRNA^Pyl^ variants were inoculated into 2 ml LB medium. The overnight culture was diluted with fresh LB medium to an absorbance of 0.2 at 600 nm, supplemented with ncAAs, 100 μg/ml ampicillin and 50 μg/ml chloramphenicol; 10 mM arabinose was added to induce the expression of sfGFP. 200 μl culture of each strain was transferred to a 96-well plate. Cells were shaken for 12 h at 37°C, with monitoring of fluorescence intensity (excitation 485 nm, emission 528 nm, bandwidths 20 nm) and optical density (A_600_, for growth curves) by plate-readers. The same strains lacking ncAAs were used as controls to measure background signals.

### Histone H3 expression and purification

The human histone H3 gene and its variants were cloned into the *pET15b* plasmid with a C-terminal His_6_-tag, and transformed into BL21(DE3) cells together with the plasmids harboring tRNAs and AckRS genes for expression. The expression strain was grown in 1 L of LB medium supplemented with 100 μg/ml ampicillin and 50 μg/ml chloramphenicol at 37°C to an absorbance of 0.6–0.8 at 600 nm, and protein expression was induced by the addition of 1 mM IPTG and supplemented with 5 mM AcK and 20 mM nicotinamine (NAM). Cells were incubated at 37°C for an additional 8 h and harvested by centrifugation at 5000 × g for 10 min at 4°C. The cell paste was suspended in 15 ml of PBS with 20 mM NAM and broken by sonication. The crude extract was centrifuged at 30000 × g for 30 min at 4°C. The pellets were washed twice with B-PER Protein Extraction Reagents (Thermo Scientific) and suspended with 20 ml of 10 mM Tris, 100 mM NaH_2_PO_4_ (pH 8), 6 M guanidinium chloride and 2 mM DTT. The samples were loaded onto a column containing previously equilibrated Ni-NTA resin (Qiagen) with with 20 ml of 10 mM Tris, 100 mM NaH_2_PO_4_ (pH 8), 8 M urea and 1 mM DTT. The column was washed with 20 ml of 10 mM Tris, 100 mM NaH_2_PO_4_ (pH 6.2), 8 M urea and 1 mM DTT. The proteins bound to the column were then eluted with 2 ml of 10 mM Tris, 100 mM NaH_2_PO_4_ (pH 4.5), 8 M urea and 1 mM DTT. The purified proteins were further analyzed by SDS-PAGE.

### Malate dehydrogenase (MDH) expression and purification

The genes of MDH and its variants were cloned into the *pET15b* plasmid with a C-terminal His_6_-tag, and transformed into BL21(DE3) cells together with the plasmids harboring tRNAs and AckRS genes for expression. The expression strain was grown on 1 l of LB medium supplemented with 100 μg/ml ampicillin and 50 μg/ml chlorampheni­col at 37°C to an absorbance of 0.6–0.8 at 600 nm, and the protein expression was induced by the addition of 1 mM IPTG and supplemented with 5 mM AcK and 20 mM NAM. Cells were incubated at 30°C for an additional 8 h, and harvested by centrifugation at 5000 × g for 10 min at 4°C. The cell paste was suspended in 15 ml of lysis buffer (50 mM Tris (pH 7.5), 300 mM NaCl, 20 mM imidazole, 20 mM NAM) and broken by sonication. The crude extract was centrifuged at 30000 × g for 30 min at 4°C. The soluble fraction was loaded onto a column contain­ing 1 ml of Ni-NTA resin (Qiagen) previously equilibrated with 20 ml lysis buffer. The column was washed with 20 ml lysis buffer. The protein bound to the column was then eluted with 2 ml of 50 mM Tris (pH 7.5), 300 mM NaCl, 200 mM imidazole. The purified protein was dialyzed with 50 mM Tris (pH 7.5), 50 mM NaCl, 1mM DTT and 50% glycerol, and stored at -80°C for further studies.

### MDH activity assay

The assays were performed by following the instruction of the EnzyChrom^TM^ Malate Dehydrogenase Assay Kit (EMDH-100) from BioAssay Systems. This non-radioactive, colorimetric MDH assay is based on the reduction of the tetrazolium salt MTT in a NADH-coupled enzymatic reaction to a reduced form of MTT which exhibits an absorption maximum at 565 nm. The increase in absorbance at 565 nm is proportional to the enzyme activity.

### LC-MS/MS analyses

The purified proteins were analyzed by LC-MS/MS. The proteins were trypsin digested by a standard in-gel digestion protocol, and analyzed by LC-MS/MS on an LTQ Orbitrap XL (Thermo Scientific) equipped with a nanoACQUITY UPLC system (Waters). A Symmetry C18 trap column (180 μm x 20 mm; Waters) and a nanoACQUITY UPLC column (1.7 μm, 100 μm x 250 mm, 35°C) were used for peptide separation. Trapping was done at 15 μl min^−1^, 99% buffer A (water with formic acid (0.1%)) for 1 min. Peptide separation was performed at 300 nl min^−1^ with buffer A and buffer B (CH_3_CN containing 0.1% formic acid). The linear gradient was from 5% buffer B to 50% B at 50 min, to 85% B at 51 min. MS data were acquired in the Orbitrap with one microscan, and a maximum inject time of 900 ms followed by data-dependent MS/MS acquisitions in the ion trap (through collision induced dissociation, CID). The Mascot search algorithm was used to search for the appropriate noncanonical substitution (Matrix Science, Boston, MA).

### tRNA synthetases expression and purification

The genes of tRNA synthetases were cloned into the *pET15a* plasmid and transformed into BL21(DE3) cells for expression. The expression strain was grown in 500 ml of LB medium supplemented with 100 μg/ml ampicillin at 37°C to an absorbance of 0.6–0.8 at 600 nm and protein expression was induced by the addition of 1 mM IPTG. Cells were incubated at 30°C for an additional 4 h and harvested by centrifugation at 5000 × g for 10 min at 4°C. The cell paste was suspended in 15 ml of lysis buffer (50 mM Tris (pH 8), 300 mM NaCl, 20 mM imidazole) and broken by sonication. The crude extract was centrifuged at 30 000 × g for 30 min at 4°C. The soluble fraction was loaded onto a column containing 1 ml of Ni-NTA resin previously equilibrated with 20 ml of lysis buffer. The column was washed with 20 ml of lysis buffer, and eluted with 2 ml of 50 mM Tris (pH 7.5), 300 mM NaCl, 200 mM imidazole. The purified protein was dialyzed with 50 mM HEPES-KOH (pH 7.5), 50 mM KCl, 1mM DTT and 50% glycerol, and stored at -80°C for further studies.

### tRNA transcription and purification

The experiments followed protocols described before (27). Template plasmids containing tRNA genes were purified with the plasmid maxi kit (Qiagen), and 100 μg of plasmid was digested with BstNI (New England Biolabs). The BstNI digested template DNA was purified by phenol chloroform extraction, followed by ethanol precipitation and resolved in double distilled water. A His_6_-tagged T7 RNA polymerase was purified over column of Ni-NTA resin according to manufacturer's instructions. The transcription reaction (40 mM Tris (pH 8); 4 mM each of UTP, CTP, GTP and ATP at pH 7.0; 22 mM MgCl_2_; 2 mM spermidine; 10 mM DTT; 6 μg pyrophosphatase (Roche Applied Science); 60 μg/ml BstNI digested DNA template, approximately 0.2 mg/ml T7 RNA polymerase) was performed in 10 ml reaction volumes for overnight at 37°C. The tRNA was purified on 12% denaturing polyacrylamide gel containing 8 M urea and TBE buffer (90 mM Tris, 90 mM boric acid, 2 mM EDTA). UV shadowing illuminates the pure tRNA band, which is excised and extracted three times with 1 M sodium acetate pH 5.3 at 4°C. The tRNA extractions were then ethanol precipitated, dissolved in RNase-free distilled water, pooled, and finally desalted using a Biospin 30 column (BioRad).

### tRNA refolding and ^32^P labeling

The tRNA was refolded by heating to 100°C for 5 min and slow cooling to room temperature. At 65°C, MgCl_2_ was added to a final concentration of 10 mM to aid folding. A His_6_-tagged CCA-adding enzyme was purified over column of Ni-NTA resin according to manufacturer's instructions (Qiagen). 16 μM refolded tRNA in 50 mM Tris (pH 8.0), 20 mM MgCl_2_, 5 mM DTT and 50 μM NaPPi was incubated at room temperature for 1 h with approximately 0.2 mg/ml CCA-adding enzyme and 1.6 μCi/μl of [α-^32^P]-labeled ATP (PerkinElmer). The sample was phenol/chloroform extracted and then passed over a Bio-spin 30 column (Bio-Rad) to remove excess ATP.

### tRNA aminoacylation assay

A 20 μl aminoacylation reaction contained the following components: 50 mM HEPES-KOH (pH 7.2), 25 mM KCl, 10 mM MgCl_2_, 5 mM DTT, 10 mM ATP, 25 μg/ml pyrophosphatase (Roche Applied Science). All tRNA aminoacylation levels were determined at 37°C with according to the reactions conditions descried above with 100 nM tRNA synthetases, 10 nM ^32^P-labeled tRNA. Time points were taken at 5 min, 10 min and 20 min by removing 2 μl aliquots from the reaction and immediately quenching the reaction into an ice-cold 3 μl quench solution (0.66 μg/μl nuclease P1 (Sigma) in 100 mM sodium citrate (pH 5.0)). For each reaction, 2 μl of blank reaction mixture (containing no enzyme) was added to the quench solution as the start time point. The nuclease P1 mixture was then incubated a room temperature for 30 min, and 1 μl aliquots were spotted on PEI-cellulose plates (Merck) and developed in running buffer containing 5% acetic acid and 100 mM ammonium acetate. Radioactive spots for AMP and AA-AMP (representing free tRNA and aminoacyl-tRNA, respectively) were separated and then visualized and quantified by phosphorimaging by a Molecular Dynamics Storm 860 phosphorimager (Amersham Biosciences). The ratio of aminoacylated tRNA to total tRNA was determined to monitor reaction progress.

### Northern blotting

The strains harboring tRNA^Pyl^ and tRNA^Pyl-opt^ were grown in LB media to an absorbance of 2.0 at 600 nm. Cells were washed with 0.3 M sodium acetate (pH 4.5), 10 mM EDTA. Total tRNA was extracted and suspended in 200 mM Tris (pH 8.0). 1 μg of total tRNA was loaded on 12% denaturing polyacrylamide gel containing 8 M urea, 1x TAE, and the gel was run at 4°C, 100V in 1x TAE buffer for 6 h. RNA was transferred onto Hybond-N+ membrane (GE Healthcare) with a Trans-Blot SD semi-dry transfer cell (Bio-Rad) at 400 mA for 40 min. RNA was cross-linked to the membrane at UVC 500 Ultraviolet Crosslinker (Amersham Biosciences) at 120 000 μJ/cm^2^. Membranes were pre-hybridized in ULTRAhyb Ultrasensitive buffer (Ambion) at 42°C for 30 min. DNA oligonucleotides complementary to tRNA^Pyl^ (TAGAGTCCATCGATCTACATG) and tRNA^Ser^_GCU_ (CTTTTGACCGCATACTCCCT) were used as probes. Probes were 5′ end labeled with [γ-^32^P]ATP and added to the hybridization (10^6^ cpm/ml) at 42°C for 16 h. The membrane was washed with 2x SSC, 0.1% SDS buffer at 42°C for 30 min, washed with 0.1x SSC, 0.1% SDS buffer at 42°C for 30 min, and visualized by phosphorimaging by a Molecular Dynamics Storm 860 phosphorimager (Amersham Biosciences).

## RESULTS

### Rationally evolving tRNA^Pyl^ to increase AcK incorporation in *E. coli*

The crystal structure of the ternary complex of *E. coli* Cys-tRNA^Cys^ and *Thermus aquaticus* EF-Tu demonstrated that several residues in the acceptor and T stems of tRNA^Cys^ interact with EF-Tu (28). Previous study also showed that the mutations in the acceptor and T stems of the tRNA could increase the incorporation of ncAAs in *E. coli* (29,30). tRNA^Pyl^ is an archaeal tRNA and may not bind optimally to *E. coli* EF-Tu. Thus we rationally evolved the acceptor stem and T stem of tRNA^Pyl^ for improved AcK incorporation.

First, we created Library I for the 2–71 and 3–70 positions in tRNA^Pyl^, replacing these two base pairs with standard base pairs (GC/CG and AU/UA) as well as one wobble pair (GU/UG), which is known to enhance tRNA binding to *E. coli* EF-Tu (24). Then we screened all these variants to identify the best tRNA to facilitate AcK incorporation in *E. coli*. For this screen we used the sfGFP reporter bearing an amber codon at the permissive position 2. The sfGFP was co-expressed with the plasmid encoding the tRNA^Pyl^ variants and AcKRS (20). The efficiency of AcK incorporation was measured by the sfGFP fluorescence intensity normalized by the corresponding cell density. The best performing tRNA in Library I turned out to be the G2-C71/A3-U70 in wild-type tRNA^Pyl^. Interestingly, any mutations at these two base pairs reduced AcK incorporation (Supplementary Table S1).

**Figure 1.**
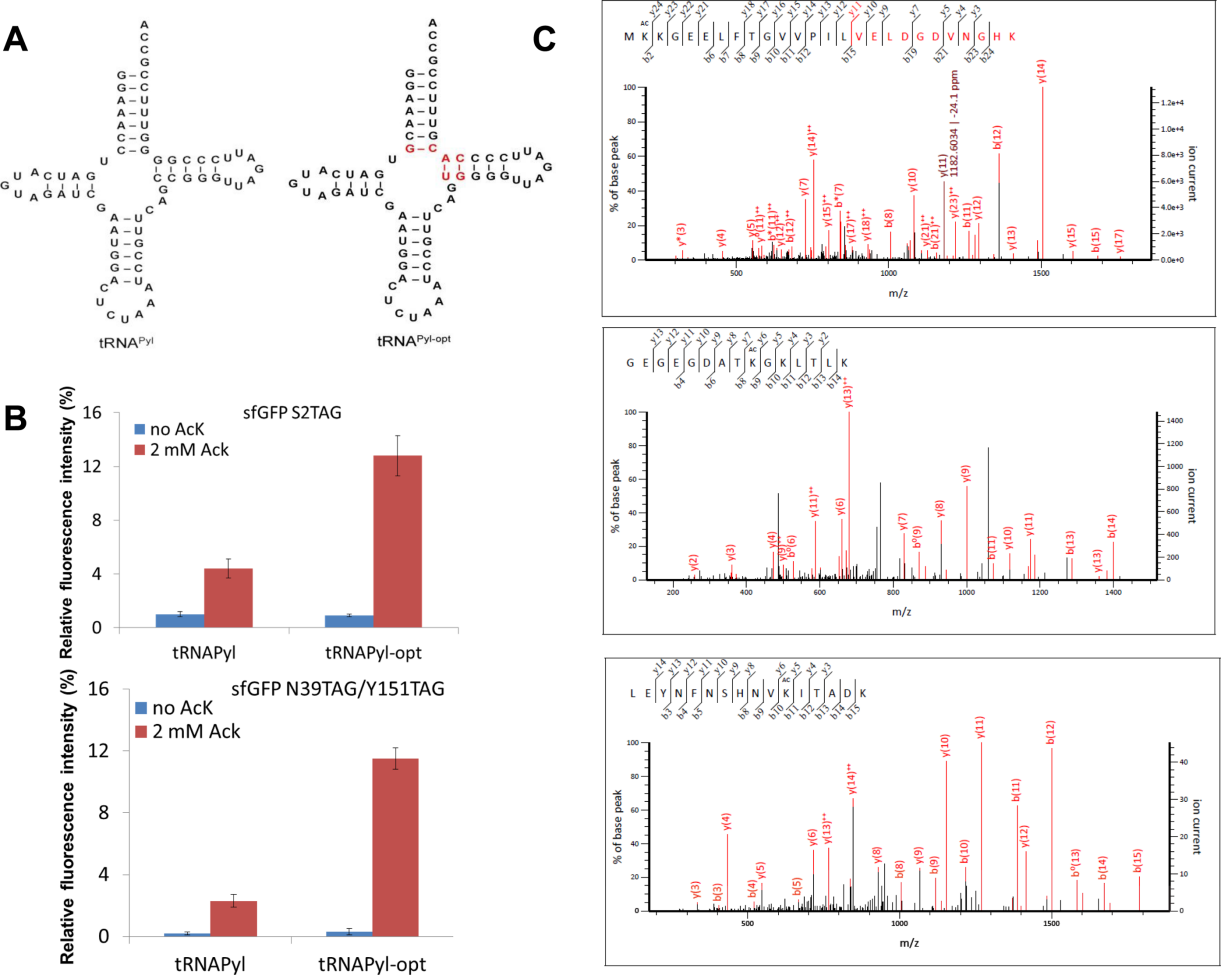
AcK incorporation in sfGFP. (**A**) The secondary structures of tRNA^Pyl^ and tRNA^Pyl-opt^. The based pairs different from the wild-type are marked with red color. (**B**) The readthrough of amber codon(s) in sfGFP by tRNA^Pyl^ and tRNA^Pyl-opt^. The relative fluorescence intensities were calculated from the absolute fluorescence intensities read at 12 h normalized by the corresponding cell densities. The values for the full-length sfGFP are set as 100%. The mean values and standard errors were calculated from three replicates. (**C**) LC-MS/MS analyses of sfGFP variants. The top one is the tandem mass spectrum of the peptide (residues 1–26) MK^Ac^KGEELFTGVVPILVELDG-DVNGHK (ion score 90) from purified sfGFP with one amber codon at position 2. The middle and bottom ones are the tandem mass spectrum of the peptides (residues 31–45) GEGEGDATK^Ac^GKLTLK (ion score 85) and (residues 141–156) LEYNFNSHNVK^Ac^ITADK (ion score 101) from purified sfGFP with two amber codons at position 39 and 151. K^Ac^ denotes AcK incorporation. The partial sequences of the peptides containing the AcK can be read from the annotated b or y ion series.

Then we created Library II for 6–67 and 7–66 base pairs with the same replacement strategy as for Library I, and measured efficiency by the same sfGFP readthrough assay. The best tRNA variant was C6-G67/G7-C66 (*versus* C6-G67:C7-G66 in wild-type tRNA^Pyl^), with 2.4-fold increased AcK incorporation (The background readings without AcK in the medium were subtracted.) (Supplementary Table S1). Keeping these best mutations we created Library III for 49–65 and 50–64 base pairs with the same replacement and screening strategy as used above. The best tRNA variant was U49-A65/G50-C64 (*versus* C49-G65/C50-G64 in wild-type tRNA^Pyl^) with a slight improvement (136%) in AcK incorporation (Supplementary Table S1). This variant, named tRNA^Pyl-opt^, facilitated a ∼3-fold increase in AcK incorporation programmed by one amber codon (Figure 1B).

Proteomic studies showed that acetylation at multiple lysine is well known in single proteins (31). To test whether tRNA^Pyl-opt^ will also improve multiple AcK incorporation we used a sfGFP gene with amber codons at the permissive positions 39 and 151 as the reporter (32). tRNA^Pyl-opt^ increased the simultaneous readthrough of these two amber codons 5.0-fold (from 2.3% suppression to 11.5% suppression, with an AcK concentration of 2 mM in the growth medium) (Figure 1B). Upon purification of the sfGFP variants by Ni-NTA affinity chromatography, LC-MS/MS analyses confirmed complete AcK incorporation at the specified positions (Figure 1C).

### Testing tRNA^Pyl-opt^ for incorporation of AcK residues in *E. coli* MDH

We chose MDH, an enzyme that plays a crucial role in important metabolic pathways such as the tricarboxylic acid cycle and glyoxylate bypass (33), as the target to measure the enhanced performance of tRNA^Pyl-opt^ over tRNA^Pyl^ by site-specifically incorporating one or both AcKs (residues 99 and 140) found naturally in this protein (34–36).

**Figure 2.**
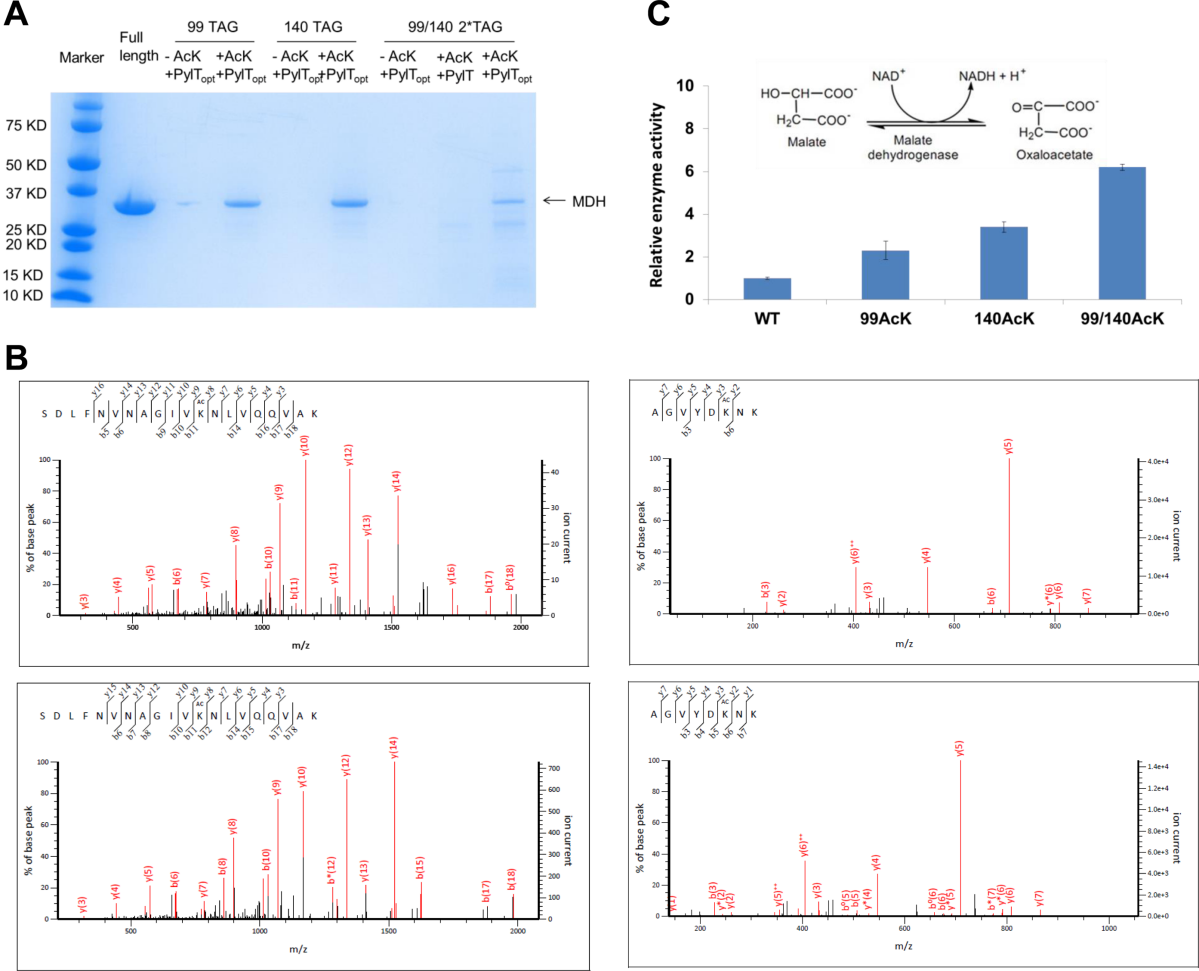
AcK incorporation in the MDH protein. (**A**) Coomassie blue stained SDS-PAGE gel of purified MDH and its acetylated variants. The same volumes of the elution were loaded on the gel. The theoretical molecular weight of MDH is 32 kD. (**B**) LC-MS/MS analyses of MDH variants. Top left: tandem mass spectrum of the peptide (residues 88–107) SDLFNVNAGIV-K^Ac^NLVQQVAK (ion score 109) from purified MDH with one amber codon at position 99. Top right: tandem mass spectrum of the peptide (residues 135–142) AGVYDK^Ac^NK (ion score 52) from purified MDH with one amber codon at position 140. The bottom ones: tandem mass spectra of the peptides (residues 88–107) SDLFNVNAGIVK^Ac^N-LVQQVAK (ion score 103) and (residues 135–142) AGVYDK^Ac^NK (ion score 48) from purified MDH with two amber codons at positions 99 and 140. K^Ac^ denotes AcK incorporation. The partial sequences of the peptides containing the AcK can be read from the annotated b or y ion series. (**C**) The enzyme activities of MDH and its acetylated variants. The mean values and standard errors were calculated from three replicates. The enzyme activity of wild-type MDH is set as 1, and the reaction catalyzed by MDH is demonstrated in the figure.

The C-terminal His_6_-tagged MDH bearing an amber codon at positions 99 or 140 was co-expressed with the tRNA^Pyl-opt^ and AcKRS genes, individually. The purified MDHs were loaded on the SDS-PAGE gel (Figure 2A). Without AcK in the growth medium, no visible band was observed. In the presence of 5 mM AcK and 20 mM NAM (to inhibit deacetylase) in the growth medium, we obtained 4.3 and 5.6 mg/l MDH/AcK99 and MDH/AcK140, respectively; the yield of wild-type MDH (lacking AcK) is 28.9 mgl under these conditions. Then we compared simultaneous incorporation of two AcK residues into positions 99 and 140 with amber codons by the wild-type tRNA^Pyl^ and tRNA^Pyl-opt^. There was no detectable band in the SDS-PAGE gel with the tRNA^Pyl^, while we were able to get 0.6 mg/l MDH protein with AcK incorporated into both positions using tRNA^Pyl-opt^. LC-MS/MS analyses confirmed complete AcK incorporation at the specified positions (Figure 2B).

Previous studies showed that lysine acetylation activates MDH activity in human cells (31,37,38), thus we tested the effect of lysine acetylation on the *E. coli* MDH. Our results showed that individual lysine acetylation at positions 99 or 140 increased MDH activities by 2.3- or 3.4-fold. When positions 99 and 140 were simultaneously acetylated, the MDH activity was 6-fold higher than that of unmodified enzyme (Figure 2C). Our results indicate that acetylation of *E. coli* MDH shares the same regulatory mechanism with different organisms. To our knowledge, this is the first study on the lysine acetylation of metabolic enzymes in *E. coil*.

### Testing tRNA^Pyl-opt^ for incorporation of AcK residues in human histone H3

Next, we chose the human histone H3, a target of earlier studies of successful AcK incorporation (39,40), as the reporter protein to assess the performance of tRNA^Pyl-opt^ versus tRNA^Pyl^ in site-specifically simultaneous incorporation of two AcK residues (positions 14 and 56), that are acetylated in the native protein (41).

**Figure 3.**
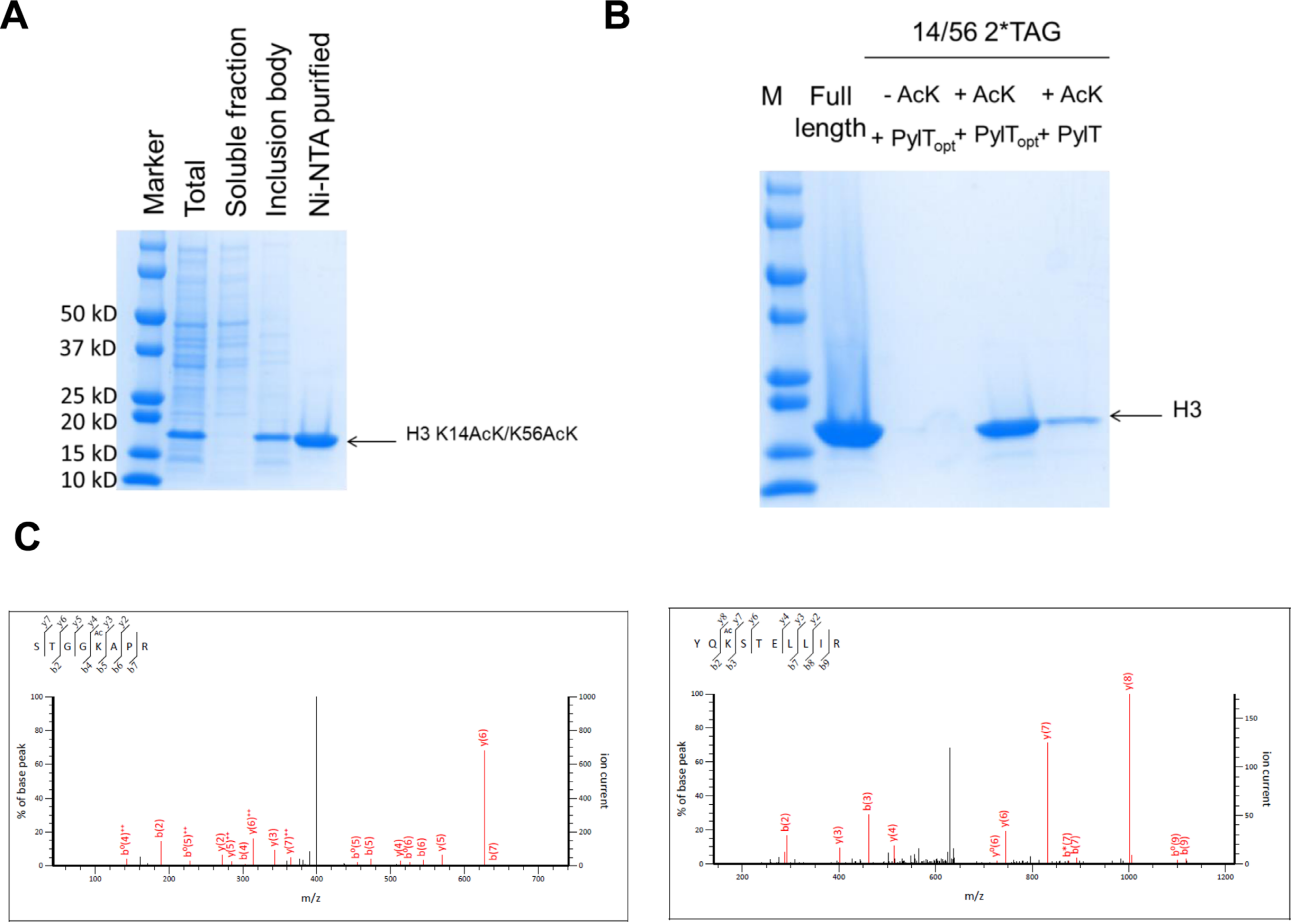
AcK incorporation in histone H3 protein. (**A**) Coomassie blue stained SDS-PAGE gel of the purification of H3 K14AcK/K56AcK. (**B**) Coomassie blue stained SDS-PAGE gel of purified H3 and its acetylated variants. The same volumes of the elution were loaded on the gel. The theoretical molecular weight of H3 is 16 kD. (**C**) LC-MS/MS analyses of H3 K14AcK/K56AcK. Left: tandem mass spectrum of the peptide (residues 10–17) STGGK^Ac^APR (ion score 59) for position 14. Right: tandem mass spectrum of the peptide (residues 54–62) YQK^Ac^TELLIR (ion score 61) for position 56. K^Ac^ denotes AcK incorporation. The partial sequences of the peptides containing the AcK can be read from the annotated b or y ion series.

The C-terminal His_6_-tagged H3 gene with two amber codons at positions 14 and 56 was co-expressed with the AcKRS•tRNA^Pyl^ or AcKRS•tRNA^Pyl-opt^ pair, individually. Cells were supplemented with 5 mM AcK and 20 mM NAM. Histone H3 and its variants were found in the inclusion bodies and purified by denaturing Ni-NTA affinity chromatography (Figure 3A). Without AcK in the growth medium, no visible band was observed. Providing 5 mM AcK in the growth medium, with tRNA^Pyl-opt^ we obtained 1.2 mg/l H3 protein containing AcK in both positions, while we only got ∼0.2 mg/l protein containing AcK by wild-type tRNA^Pyl^ (The yield of full length H3 protein is 22 mg/l in the same growth medium) (Figure 3B). LC-MS/MS analyses confirmed complete AcK incorporation at the specified positions (Figure 3C).

### tRNA^Pyl-opt^ increased the incorporation of other ncAAs by PylRS and its variants

**Figure 4.**
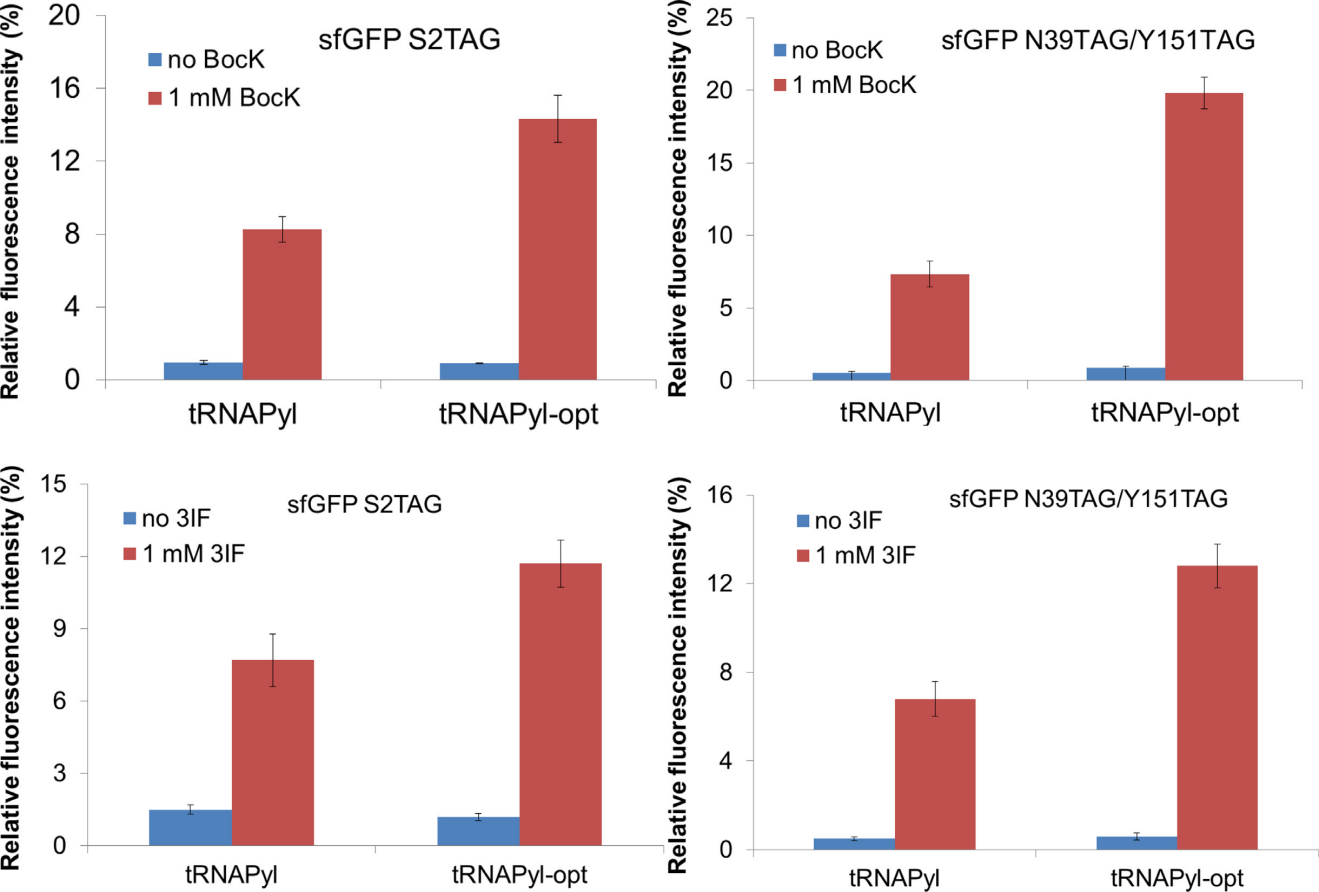
The improved incorporation of ncAAs by PylRS and IFRS. The sfGFP with one amber codon at position 2 and the sfGFP with two amber codons at positions 39 and 151 were used as reporters. The upper panels are for wild-type PylRS with BocK, and the lower panels are for IFRS with 3-iodo-Phe. The relative fluorescence intensities were calculated from the absolute fluorescence intensities read at 12 h normalized by the corresponding cell densities. The values for the full-length sfGFP are set as 100%. The mean values and standard errors were calculated from three replicates.

Previous studies showed that tRNAs charged with non-cognate amino acids have a broad range of affinities for EF-Tu (42,43). Thus we determined the effect of tRNA^Pyl-opt^ on other ncAAs incorporation by the wild-type PylRS and a PylRS variant (IFRS) evolved for 3-iodo-Phe (22). Here, we used BocK as the efficient substrate for wild-type PylRS (11). sfGFP with one amber codon at position 2, or with two amber codons at positions 39 and 151 were the reporter proteins. They were co-expressed with the plasmid encoding the tRNAs and tRNA synthetases, respectively. For BocK incorporation by PylRS, the readthrough of one amber codon increased by 1.7-fold while the readthrough of two amber codons increased by 2.7-fold. For 3-iodo-Phe incorporation by IFRS, the readthrough of one amber codon increased by 1.5-fold while the readthrough of two amber codons increased by 1.9-fold (Figure 4).

**Figure 5.**
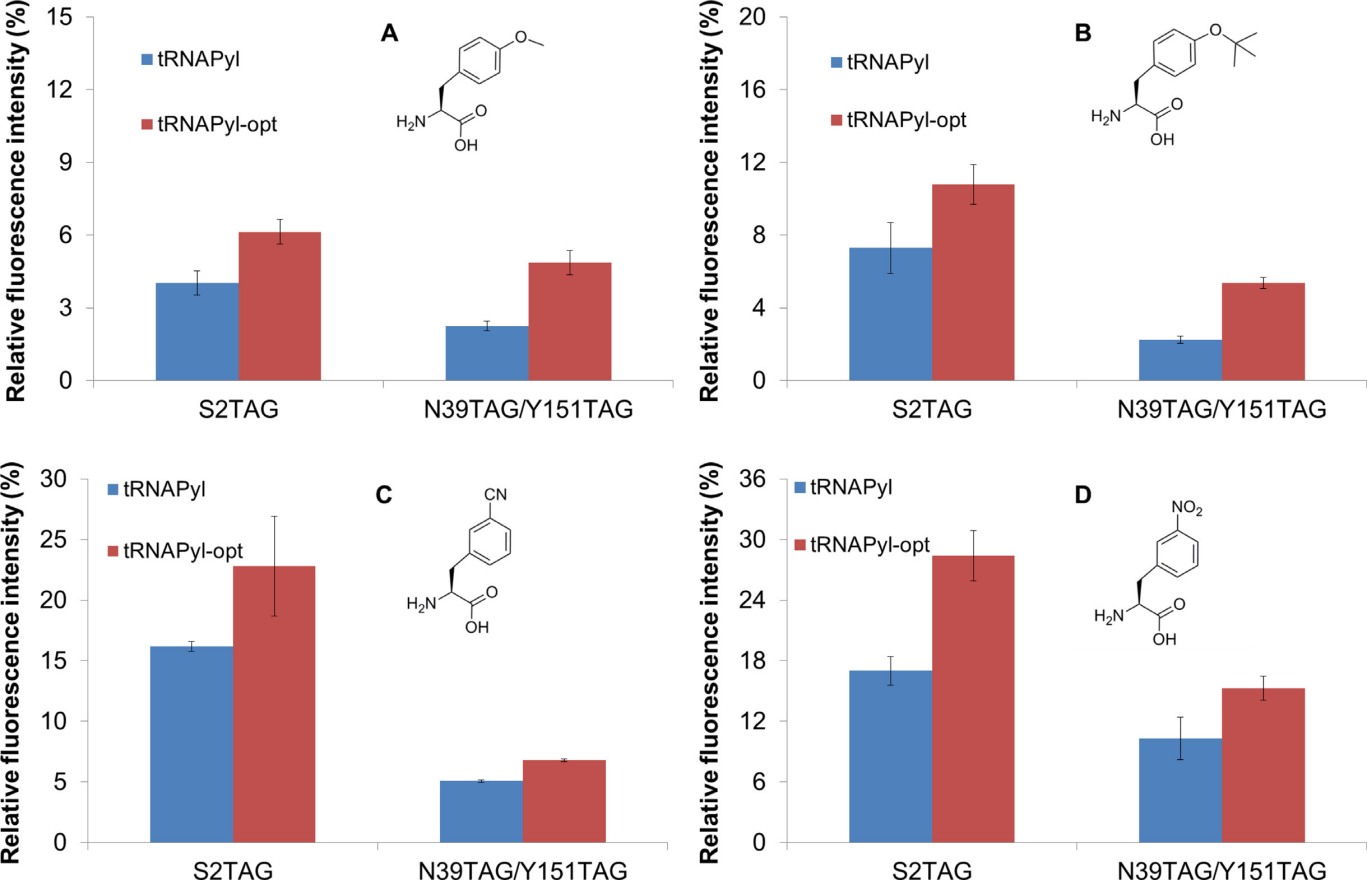
The improved incorporation of four ncAAs by one broad specific PylRS variant. The ncAAs used were (**A**) *O*-methyl-tyrosine, (**B**) *O-tert*-butyl-tyrosine, (**C**) 3-cyano-phenylalanine and (**D**) 3-nitro-phenylalanine with 2 mM in the growth medium, individually. The relative fluorescence intensities were calculated from the absolute fluorescence intensities read at 12 h normalized by the corresponding cell densities. The values for the full-length sfGFP are set as 100%. The mean values and standard errors were calculated from three replicates. The background with no ncAAs provided in the medium was subtracted in each experiment.

To further explore the enhanced effect of tRNA^Pyl-opt^, we chose another PylRS variant (PylRS-AA) which facilitates incorporation of a broad range of ncAAs (44,45) using the same sfGFP reporters as used above. tRNA^Pyl-opt^ increased ncAA incorporation by 1.3–1.7-fold for one amber codon, and 1.4–2.4-fold for two amber codons (Figure 5). The results indicate that the incorporation of all the ncAAs tested was improved by the optimized tRNA^Pyl-opt^.

### The aminoacylation of tRNAs by PylRS enzymes

To determine substrate properties of tRNA^Pyl-opt^ we examined the aminoacylation kinetics using [^32^P]-labeled tRNAs (46) (Table 1). We tested wild-type *M. barkeri* PylRS (*Mb*PylRS) and two of PylRS variants, AcKRS and IFRS. The results showed no significant difference in the *k*_cat_ and *K*_M_ values between tRNA^Pyl^ and tRNA^Pyl-opt^. Moreover, northern blotting showed tRNA^Pyl-opt^ appears not to change the expression level and stability in *E.coli* (Supplementary Figure S1). Thus tRNA^Pyl-opt^ will be an excellent replacement of wild-type tRNA^Pyl^ in any future incorporation strategy.

**Table 1.** Apparent kinetic parameters of tRNA aminoacylation by PylRS and its variants

tRNA Synthetase	tRNA	*k*_cat_ (10^−3^ s^−1^)	*K*_M, AA_ (mM)	*K*_M, tRNA_ (μM)
*Mb*PylRS	tRNA^Pyl^	11.44 ± 0.24	1.03 ± 0.05	0.26 ± 0.07
*Mb*PylRS	tRNA^Pyl-opt^	10.78 ± 0.34	1.10 ± 0.12	0.22 ± 0.02
AcKRS	tRNA^Pyl^	6.72 ± 0.09	41 ± 7	0.21 ± 0.03
AcKRS	tRNA^Pyl-opt^	6.37 ± 0.11	39 ± 6	0.24 ± 0.08
IFRS	tRNA^Pyl^	5.02 ± 0.21	0.41 ± 0.01	0.25 ± 0.04
IFRS	tRNA^Pyl-opt^	4.86 ± 0.07	0.47 ± 0.06	0.29 ± 0.11

The mean values and standard errors were calculated from three replicates.

We also measured the aminoacylation kinetics of tRNA^Pyl^ variants that showed decreased ncAA incorporation (Supplementary Table S2). All the variants tested had no obvious effects on the catalytic efficiency and amino acid binding. The mutations at 2–71 and 3–70 base pairs had increased *K*_M_ values for tRNA indicating weaker tRNA binding with PylRS, while the mutations at 6–67 and 7–66 base pairs had no significant effects on tRNA binding with PylRS.

## DISCUSSION

Although more than 100 ncAAs have been incorporated into proteins from bacteria to mammals, many challenges remain. The most critical problem is the poor catalytic efficiency of engineered aaRSs (2). However, optimization of tRNA synthetases needs the creation of large mutant libraries as well as repeated cycles of selection which are laborious. Here, we demonstrated an facile approach to increase ncAA incorporation by improving the ‘quality’ of tRNA^Pyl^. Interestingly, a recent study of evolving tRNA^Sec^ for efficient EF-Tu mediated incorporation of selenocysteine in *E. coli* resulted in mutations in the same base-pairs of the tRNA frame (30). This convergence provides a general scheme to optimize tRNAs for different orthogonal pairs that may be needed in *E. coli*.

From Supplementary Table S1, we could see the optimal effect of tRNA^Pyl-opt^ is mainly from the inversion of the pair C7-G66 to G7-C66. All the mutants in Library II bearing G7-C66 have higher AcK incorporation (1.34–2.36-fold). And all the mutants in Library III bearing U49-A65 has minor improvement (1.10–1.36-fold) except for the combination with C50–64 (32% lower efficiency), while U50-A64 (1.34-fold) has similar efficiency with G50-C64 mutations (1.36-fold) in tRNA^Pyl-opt^. We also noticed that tRNA^Pyl-opt^ retained the 2–71 and 3–70 base pairs, and all the mutations of these residues decreased the ncAA incorporation (Supplementary Table S1). These positions do not contribute significantly to binding with EF-Tu (24), and the kinetic data of tRNA aminoacylation suggested a role in the binding with PylRS (Supplementary Table S2). When the A3-U70 pair is mutated to U3-A70, the AcK incorporation is decreased by 31% and the *K*_M_ for tRNA is ∼2-fold higher than that of wild-type tRNA^Pyl^. A tRNA^Pyl^, where the G2-C71 pair is flipped, shows a 4-fold higher *K*_M_ for tRNA and leads to a 68% decrease in AcK incorporation. The tRNA^Pyl^ variant with C2-G71/U3-A70 mutations (G2-C71/A3-U70 in wild-type) decreased the AcK incorporation by >6-fold, indicating a preference of the base size of the tRNA residues for binding with PylRS. A crystal structure of the *Desulfitobacterium hafniense* PylRS (*Dh*PylRS) complex with tRNA^Pyl^ showed that *Dh*PylRS interacts with residues 69–72 at the acceptor stem of *Dh*tRNA^Pyl^ (47). Although *Dh*PylRS corresponds only to the C-terminal catalytic core of PylRS from *Methanosarcinales*, the binding of tRNA^Pyl^ and PylRS may share the same pattern between the bacterial and archaeal pairs (48).

A previous report showed the GU pair at the T-stem of tRNA increased tRNA binding to EF-Tu (24). Yet our results indicate that for tRNA^Pyl^ the GU pair decreases the AcK incorporation. When the C6-G67 pair is mutated to U6-G67, the AcK incorporation is decreased by 21% and when the C7-G66 pair is mutated to U7-G66, the AcK incorporation is lower by 47%. The aminoacylation kinetics showed that these GU pair mutations do not affect tRNA binding with PylRS (Supplementary Table S2). Given that EF-Tu is the universal carrier for all the canonical aminoacyl-tRNAs, it is logic that the nucleotide sequences for optimal EF-Tu binding are different.

Taken together, the data discussed above support the conclusion that the evolved tRNA^Pyl-opt^ will enhance incorporation of the large variety of ncAAs that are charged by PylRS enzymes.

## Supplementary Material

SUPPLEMENTARY DATA
