# Supplementary material for: Rationally evolving tRNAPyl for efficient incorporation of noncanonical amino acids
Source: Nucleic Acids Res. 2015 Aug 6;43(22):e156. doi: 10.1093/nar/gkv800 (PMC4678846; doi:10.1093/nar/gkv800)
Supplement: SUPPLEMENTARY DATA [file supp_43_22_e156__index.html]

Rationally evolving tRNAPyl for efficient incorporation of noncanonical amino acids — SUPPLEMENTARY DATA 

# Rationally evolving tRNAPyl for efficient incorporation of noncanonical amino acids

## SUPPLEMENTARY DATA

- SUPPLEMENTARY DATA
