# Supplementary material for: Rationally evolving tRNAPyl for efficient incorporation of noncanonical amino acids
Source: Nucleic Acids Res. 2015 Aug 6;43(22):e156. doi: 10.1093/nar/gkv800 (PMC4678846; doi:10.1093/nar/gkv800)
Supplement: SUPPLEMENTARY DATA [file supp_gkv800_nar-01468-met-k-2015-File002.pdf]

Rationally evolving tRNA^{Pyl} for efficient incorporation of noncanonical amino acids

Chenguang Fan*[1], Hai Xiong[1], Noah Reynolds[1], and Dieter Söll*[1,2]

Supplementary Data

Table S1. The relative AcK incorporation efficiency by tRNA^{Pyl} variants

Library I		Library II		Library III	
Mutations	Efficiency*	Mutations	Efficiency	Mutations	Efficiency
**G2-C71/A3-U70**	1.00 ± 0.04	C6-G67/C7-G66	1.00 ± 0.07	C49-G65/C50-G64	1.00 ± 0.04
G2-C71/G3-U70	0.88 ± 0.07	C6-G67/U7-A66	1.17 ± 0.10	C49-G65/U50-A64	1.01 ± 0.03
G2-C71/G3-C70	0.85 ± 0.12	C6-G67/U7-G66	0.53 ± 0.06	C49-G65/U50-G64	0.94 ± 0.11
G2-C71/C3-G70	0.74 ± 0.02	C6-G67/A7-U66	1.26 ± 0.01	C49-G65/A50-U64	0.97 ± 0.00
G2-C71/U3-A70	0.69 ± 0.10	C6-G67/G7-U66	0.60 ± 0.08	C49-G65/G50-U64	0.81 ± 0.05
G2-C71/U3-G70	0.72 ± 0.09	**C6-G67/G7-C66**	**2.36 ± 0.15**	C49-G65/G50-C64	0.78 ± 0.03
G2-U71/A3-U70	0.60 ± 0.08	G6-U67/A7-U66	0.80 ± 0.02	G49-U65/A50-U64	0.67 ± 0.04
G2-U71/G3-U70	0.62 ± 0.04	G6-U67/G7-U66	0.22 ± 0.00	G49-U65/G50-U64	0.52 ± 0.07
G2-U71/G3-C70	0.48 ± 0.05	G6-U67/G7-C66	1.34 ± 0.15	G49-U65/G50-C64	0.72 ± 0.10
G2-U71/C3-G70	0.46 ± 0.03	G6-U67/C7-G66	0.60 ± 0.05	G49-U65/C50-G64	0.60 ± 0.00
G2-U71/U3-A70	0.34 ± 0.06	G6-U67/U7-A66	0.74 ± 0.11	G49-U65/U50-A64	0.79 ± 0.05
G2-U71/U3-G70	0.50 ± 0.12	G6-U67/U7-G66	0.20 ± 0.01	G49-U65/U50-G64	0.53 ± 0.09
C2-G71/A3-U70	0.32 ± 0.01	G6-C67/A7-U66	1.10 ± 0.03	G49-C65/A50-U64	1.09 ± 0.01
C2-G71/G3-U70	0.30 ± 0.02	G6-C67/G7-U66	0.58 ± 0.02	G49-C65/G50-U64	0.90 ± 0.10
C2-G71/G3-C70	0.23 ± 0.04	G6-C67/G7-C66	1.82 ± 0.08	G49-C65/G50-C64	0.64 ± 0.02
C2-G71/C3-G70	0.18 ± 0.02	G6-C67/C7-G66	0.84 ± 0.04	G49-C65/C50-G64	1.01 ± 0.04
C2-G71/U3-A70	0.17 ± 0.06	G6-C67/U7-A66	1.69 ± 0.11	G49-C65/U50-A64	1.14 ± 0.05
C2-G71/U3-G70	0.21 ± 0.08	G6-C67/U7-G66	0.62 ± 0.07	G49-C65/U50-G64	0.89 ± 0.07
A2-U71/ A3-U70	0.53 ± 0.10	A6-U67/ A7-U66	0.83 ± 0.04	A49-U65/A50-U64	0.97 ± 0.10
A2-U71/ G3-U70	0.62 ± 0.09	A6-U67/ G7-U66	0.42 ± 0.02	A49-U65/G50-U64	1.01 ± 0.03
A2-U71/ G3-C70	0.45 ± 0.11	A6-U67/ G7-C66	1.45 ± 0.15	A49-U65/G50-C64	1.14 ± 0.01
A2-U71/ C3-G70	0.36 ± 0.07	A6-U67/ C7-G66	0.76 ± 0.04	A49-U65/C50-G64	0.79 ± 0.00
A2-U71/ U3-A70	0.40 ± 0.08	A6-U67/ U7-A66	1.08 ± 0.11	A49-U65/U50-A64	1.06 ± 0.17
A2-U71/ U3-G70	0.37 ± 0.06	A6-U67/ U7-G66	0.40 ± 0.06	A49-U65/U50-G64	0.98 ± 0.08
U2-A71/ A3-U70	0.36 ± 0.02	U6-A67/ A7-U66	1.36 ± 0.10	U49-A65/A50-U64	1.22 ± 0.03
U2-A71/ G3-U70	0.37 ± 0.04	U6-A67/ G7-U66	0.57 ± 0.02	U49-A65/G50-U64	1.10 ± 0.10
U2-A71/ G3-C70	0.26 ± 0.06	U6-A67/ G7-C66	2.26 ± 0.18	**U49-A65/G50-C64**	**1.36 ± 0.01**
U2-A71/ C3-G70	0.19 ± 0.07	U6-A67/ C7-G66	0.99 ± 0.04	U49-A65/C50-G64	0.68 ± 0.00
U2-A71/ U3-A70	0.10 ± 0.01	U6-A67/ U7-A66	0.90 ± 0.05	U49-A65/U50-A64	1.34 ± 0.13
U2-A71/ U3-G70	0.13 ± 0.00	U6-A67/ U7-G66	0.53 ± 0.09	U49-A65/U50-G64	1.19 ± 0.02
U2-G71/ A3-U70	0.41 ± 0.04	U6-G67/ A7-U66	0.91 ± 0.02	U49-G65/A50-U64	0.78 ± 0.07
U2-G71/ G3-U70	0.46 ± 0.08	U6-G67/ G7-U66	0.56 ± 0.04	U49-G65/G50-U64	0.54 ± 0.05
U2-G71/ G3-C70	0.36 ± 0.15	U6-G67/ G7-C66	1.36 ± 0.09	U49-G65/G50-C64	1.03 ± 0.14
U2-G71/ C3-G70	0.39 ± 0.09	U6-G67/ C7-G66	0.79 ± 0.14	U49-G65/C50-G64	0.84 ± 0.02
U2-G71/ U3-A70	0.27 ± 0.02	U6-G67/ U7-A66	0.76 ± 0.09	U49-G65/U50-A64	0.90 ± 0.10
U2-G71/ U3-G70	0.31 ± 0.03	U6-G67/ U7-G66	0.51 ± 0.01	U49-G65/U50-G64	0.67 ± 0.09

* The relative efficiency was compared with the values for wild-type tRNA^{Pyl} in each library which are listed in the first line. The best variants for each library are in bold. All the variants in Library III have the same C6-G67/G7-C66 mutations. The mean values and standard errors were calculated from three replicates.

Table S2. Apparent kinetic parameters of aminoacylation of tRNA variants by PylRS.

	k_{cat} (10^{-3} s^{-1})	$K_{M, BocK}$ (mM)	$K_{M, tRNA}$ (μM)
$tRNA^{Pyl}$	11.44 ± 0.24	1.03 ± 0.05	0.26 ± 0.07
$tRNA^{Pyl}$ (C2-G71)	10.57 ± 0.12	1.00 ± 0.10	1.15 ± 0.21
$tRNA^{Pyl}$ (U3-A70)	11.01 ± 0.21	1.07 ± 0.04	0.56 ± 0.10
$tRNA^{Pyl}$ (U6-G67)	11.23 ± 0.07	0.98 ± 0.07	0.23 ± 0.08
$tRNA^{Pyl}$ (U7-G66)	11.10 ± 0.19	0.93 ± 0.11	0.28 ± 0.03

The mean values and standard errors were calculated from three replicates.

Figure S1

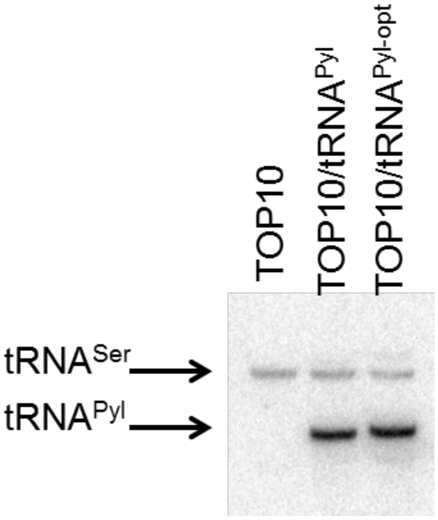

Figure S1. Northern Blots for tRNA expression and stability in *E. coli*. Total tRNA extractions from strains harboring $tRNA^{Pyl}$ and $tRNA^{Pyl\text{-}opt}$ were loaded in 12% denaturing polyacrylamide gel. Top10 without vectors was used as the negative control. *E. coli* $tRNA^{Ser}_{GCU}$ was chosen as the internal control.
